# Exposure of *Mytilus galloprovincialis* to Microplastics: Accumulation, Depuration and Evaluation of the Expression Levels of a Selection of Molecular Biomarkers

**DOI:** 10.3390/ani14010004

**Published:** 2023-12-19

**Authors:** Federica Pizzurro, Eliana Nerone, Massimo Ancora, Marco Di Domenico, Luana Fiorella Mincarelli, Cesare Cammà, Romolo Salini, Ludovica Di Renzo, Federica Di Giacinto, Corinne Corbau, Itana Bokan, Nicola Ferri, Sara Recchi

**Affiliations:** 1 Istituto Zooprofilattico Sperimentale dell’Abruzzo e Molise (IZSAM), 64100 Teramo, Italy; f.pizzurro@izs.it (F.P.); m.ancora@izs.it (M.A.); m.didomenico@izs.it (M.D.D.); c.camma@izs.it (C.C.); r.salini@izs.it (R.S.); l.direnzo@izs.it (L.D.R.); f.digiacinto@izs.it (F.D.G.); n.ferri@izs.it (N.F.); s.recchi@izs.it (S.R.); 2 Dipartimento di Scienze dell’Ambiente e della Prevenzione, Università di Ferrara, 44122 Ferrara, Italy; cbc@unife.it; 3 Teaching Institute of Public Health (TIPH), 51000 Rijeka, Croatia; itanabokan@yahoo.co.uk

**Keywords:** *Mytilus galloprovincialis*, shellfish, microplastics, depuration, marine pollution

## Abstract

**Simple Summary:**

Microplastics are an environmental pollutant increasingly present in seawater, the spread of which also represents a threat to food safety. In fact, these particles can be ingested through various foods, among the most at risk are bivalve molluscs, as they filter large quantities of seawater and enter the diet of consumers ingested entirely. Purification studies of bivalves could allow us to understand in a more precise way the ability of organisms to eliminate microplastics, in order to test this process as a potential method of removing such contaminants from bivalves intended for human consumption.

**Abstract:**

Microplastic contamination is a growing marine environmental issue with possible consequences for seafood safety. Filter feeders are the target species for microplastic (MPs) pollution because they filter large quantities of seawater to feed. In the present study, an experimental contamination of *Mytilus galloprovincialis* was conducted using a mixture of the main types of MPs usually present in the seawater column (53% filaments, 30% fragments, 3% granules) in order to test the purification process as a potential method for removing these contaminants from bivalves intended for human consumption. A set of molecular biomarkers was also evaluated in order to detect any variations in the expression levels of some genes associated with biotransformation and detoxification, DNA repair, cellular response, and the immune system. Our results demonstrate that: (a) the purification process can significantly reduce MP contamination in *M. galloprovincialis*; (b) a differential expression level has been observed between mussels tested and in particular most of the differences were found in the gills, thus defining it as the target organ for the use of these biomarkers. Therefore, this study further suggests the potential use of molecular biomarkers as an innovative method, encouraging their use in next-generation marine monitoring programs.

## 1. Introduction

Global ever-increasing production of plastic is expected to reach 33 billion tons by 2050, and they are constantly poured out in terrestrial and aquatic environments worldwide. Plastic debris, as a result of their fragmentation, produces microplastics (MPs), plastic particles with a diameter of less than 5 mm. MPs, to date, are extensively recognized as ubiquitous contaminants in aquatic environments [1,2,3,4,5] and at the same time as a worrying contaminant for human health [6,7,8,9]. Indeed, these particles easily disperse in the seawater column and are frequently found in marine biota [10,11,12]. Moreover, MPs have a high capacity for adsorbing organic pollutants from surrounding water, which can then be released into the organisms upon ingestion [13,14,15,16]. Benthic filter feeders, such as mussels, are prone to ingest microplastic particles [17,18,19,20,21,22] due to their filtering capacity allowing them to feed on planktonic organisms that have a similar size to MPs [6]. Indeed, several studies highlight the presence of MPs in many species of filter-feeding bivalves [23,24,25,26,27]. Among the detected MPs in bivalves, fibers are the most abundant independent of the location and species [28,29].

Mussels, i.e., *Mytilus galloprovincialis*, are an excellent species both as sentinel organisms in MPs pollution monitoring and for MPs experimental studies. Indeed, they are filter-feeding organisms worldwide distributed [24], very tolerant to salinity changes and other stressors, and also able to accumulate particulate pollutants, having a high-water filtration rate and low metabolic activity [12,30].

Specifically, MPs enter bivalves through the gills, being the first entry point for particulate pollutants and associated chemical and microbiological contaminants, then move towards the mouth and enter into the digestive gland [31,32,33]. These two organs have been the subject of some recent studies related to biomolecular biomarkers, which were intended to detect gene expression variations caused by pollutants’ exposure, including MPs, in aquatic organisms [34,35,36].

Although to date there are several papers related to the presence of MPs in bivalves there is still a lack of data and more detailed studies are needed about their depuration capacity. The depuration process, which consists of placing bivalves in clean seawater in an aquarium where filtration rates are maximized, reduces contaminant levels in these organisms [37].

This practice is commonly used in the shellfish aquaculture industry to remove microbiological contaminants such as *Escherichia coli* from bivalves cultivated in areas where such microbes might occur in harmful amounts [5]. Similarly, it has been demonstrated that this technique could be able to reduce the presence of MPs from bivalves [33,38,39,40,41,42,43]. It is, therefore, necessary to examine in depth the use of this practice in the shellfish purification centers (CDM) in order to release on the market a product as healthy as possible.

The present work aims to study accumulation and depuration in *M. galloprovincialis* after exposure of bivalves to known concentrations of an MP’s mixture under controlled conditions, in order to test the depuration process as a potential method for removing MPs from bivalves intended for human consumption. Furthermore, tested mussels’ digestive glands and gills were examined by reverse transcription quantitative PCR (RT-qPCR) to evaluate the gene expression levels of a selection of molecular biomarkers. These indicators are usually involved in bivalves’ response to stress due to microplastic pollutants and specifically are associated with different processes such as biotransformation and detoxification (cytochrome P450-3-like-2, cytochrome P450-1-like-1, π-glutathione-S-transferase), DNA repair (tumor protein, p53), cellular response (heat shock protein 70), and innate immunity (cathepsin and lysozyme).

## 2. Materials and Methods

### 2.1. Experimental Design

The experiments were conducted at the laboratories of Marine Ecosystem and Fisheries Centre of the Istituto Zooprofilattico Sperimentale dell’Abruzzo e del Molise “G. Caporale”, in the NET4mPLASTIC project [44]. Although we have treated animals that do not require authorization by an ethical committee for animal testing, we have however asked for information from the Ethics Committee of University of Teramo (Italy), which declared that our experiment was out of Directive 63/2010 of the European Parliament and of the Council on the protection of animals used for scientific purposes (transposed into Italian law by Legislative Decree 26/2014).

**Figure 1:**
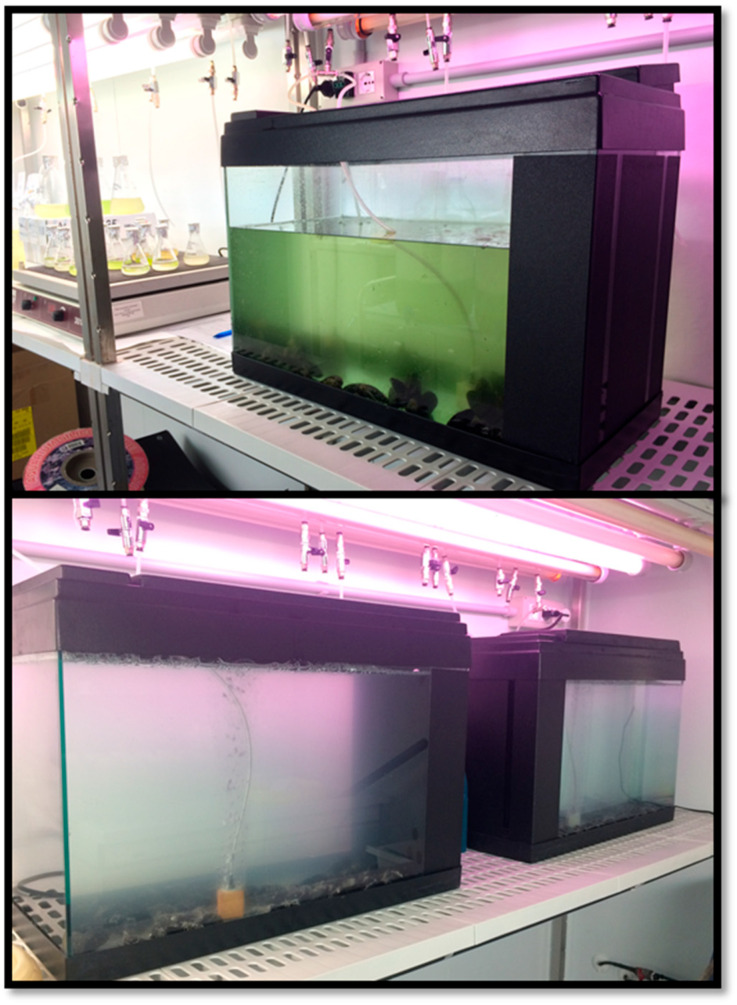
Experimental setup including the two 50 L glass aquariums containing filtered artificial seawater, placed in thermostatic chamber at 20 °C, with inside mussels tested.

Mussels (total number: 360 organisms) used for the experiments of exposure to MPs and depuration were collected from the Defmar mussel farm located at Termoli (Italy), selected based on the commercial size class, i.e., 4–7 cm in shell height. Three replicates were conducted, and each experimental group was made up of 120 individuals (divided into 2 groups of 60). The experimental setup consisted of two 50 L glass aquariums containing filtered artificial seawater (Instant Ocean; 0.8 mm membrane filter, Supor^®^ 800) placed in a climatic room at constant temperature of 18 ± 1 °C. (Figure 1).

The experimental protocol lasted 17 days and it included the following consequential phases: (a) acclimatization (7 days) (control group), (b) exposure phase (3 days) (T0 group), (c) 2 days depuration (T2 group), and d) 7 days depuration (T7 group). At the end of each phase, 20 mussels were collected (10 for each aquarium) for MPs’ qualitative–quantitative analysis (see Section 2.3). Moreover, 5 mussels were sampled for gene expression analysis (see Section 2.4).

First, once arrived in the laboratory, mussels were scrubbed to remove biofouling and then acclimated for 7 days in two 50 L glass aquarium systems containing filtered artificial seawater. During this acclimatization phase, mussels were maintained under photoperiod regime of 12 h light–12 h dark, and water’s chemical–physical parameters were monitored (temperature of 18 ± 1 °C, salinity 32–35‰, dissolved oxygen ≥80%, and pH 7.5–8.5). Furthermore, filtered artificial seawater used was continuously aerated and changed daily. No food was supplied for the entire duration of acclimatization. This phase is necessary to allow the clearance of the mussels’ gut. After the acclimatization period, 20 mussels (control group—not treated mussels) were collected for the digestion and MPs’ qualitative–quantitative analysis, while the remaining organisms were used for a 3-day exposure phase.

For the exposure phase, three main types of microplastics, usually found in seawater columns, were used: 53% fibers, 30% fragments, and 3% granules [45], with 10^4^ particles/L as frequently reported in marine environment [46] (see the following paragraph 2.2). The experiments were performed at constant salinity of 32–35‰, temperature of 18 ± 1 °C, and a 12 h light–dark regime. The mortality of mussels was monitored daily, and the water was renewed to ensure that previously ingested material, including microplastic particles, would not be ingested again.

Furthermore, during the whole period, mussels were fed daily with algal cultures of *Isochrysis galbana* and *Tetraselmis suecica* with a dose equal to 3% of mussels’ dry weight, ration considered enough to fulfill mussels’ daily energy requirements [47]. The algae were cultured using filtered seawater starting from small volume (50 mL) until massive algal production (150–200 L), via intermediate volumes. Before feeding, algal cell densities were measured using Bürker chamber, to ensure the right concentration to add to each experimental tank.

At the end of the 3-day exposure phase, mussels were removed from the exposure tanks and thoroughly rinsed to avoid any transfer of microplastics. Twenty mussels (0-time group) were removed and prepared for digestion and MPs’ qualitative–quantitative analysis, while other organisms were moved to another tank with clean seawater and subsequently underwent depuration process.

Depuration phase lasted a total of 7 days during which monitoring of mortality, water renewal, and feeding of organisms was carried out on a daily basis. At the end of the 2-day depuration period, 20 mussels (2-time group) were removed and prepared for digestion and MPs’ qualitative–quantitative analysis, while other organisms continued purification for up to 7 days. At the end of the 7-day depuration period, 20 mussels (7-time group) were removed and prepared for digestion and MPs’ qualitative–quantitative analysis. Two different depuration times were chosen:

(a) A “microbiological” depuration lasting 2 days, corresponding to the time usually applied for microbiological depuration in shellfish purification plants;
(b) An “experimental” depuration lasting 7 days, assuming that increasing the dwell time in the shellfish purification plants could allow a better depuration from these contaminants. The same experiment in its entirety was repeated in triplicate.

Furthermore, considering that airborne particle contamination in laboratories can be very high if precautions are not taken [48], laboratory access was restricted to researchers who wore distinctive (for subsequent particle identification) blue cotton coveralls whenever working in the room. The same attire was worn during sample processing.

### 2.2. Preparation of the Microplastic Mixture

Microplastics used for the exposure phase of the *M. galloprovincialis* samples were polystyrene (EPS) granules with a diameter of 100 and 200 µm, polypropylene (PP) filaments with a size range from 50 to 4000 µm, and polyethylene terephthalate (PET) fragments with a size range from 2 to 300 µm.

Filaments and fragments were industrial by-products; therefore, to know the number of particles present in 1 mg, we weighed, ten replicates of 1 mg of particles, and after we counted them with a stereomicroscope. Instead, granules were purchased (ChromoSphere Dry Dyed Polymer Particles; ThermoScientific™ Waltham, MA, USA) with a certification regarding their concentration.

Defined the concentration of microplastics (200 filaments/g; 300 fragments/g and 2.2 × 10^6^ granules/g), 1 L stock suspensions of the three polymers’ mixture, inside glass bottles, were prepared in filtered sea water with a concentration of 5 × 10^5^ particles/L.

Lastly, in order to obtain the MPs mixture’s final concentration of 10^4^ particles/L in the aquariums, 1 L of stock solution was poured daily for the total duration of 3 days of the contamination phase, after each water replacement.

### 2.3. MPs’ Qualitative-Quantitative Analysis

Before their dissection, mussel height (cm) was measured. Digestion of mussel’s soft body was performed for each individual separately by filling a glass bottle with 20 mL of 15% H_2_O_2_ per gram of soft tissues, according to the Mathalon and Hill procedure [49] and Bessa et al. [50] with minor modifications (digestion time increased until 7 days; elimination of density separation by NaCl′s phase). Bottles with samples were covered and placed in an incubator at 60–65 °C for 5–7 days.

For each experimental group, three blank samples (consisting of water plus 15% H_2_O_2_) were also performed using the same analytical methods. Blank correction was made by subtracting the mean MP particle for each size, shape, and color counted in the blanks from those found in the matching samples.

Following digestion, each sample was vacuum filtered through 47 mm diameter, 2.7 µm pore size glass microfiber filters (Whatman^®^ glass microfiber filters, Grade GF/D, GE HealthCare, Chicago, IL, USA). The filters thus obtained were then placed in glass Petri dishes and left to dry at room temperature.

**Figure 2:**
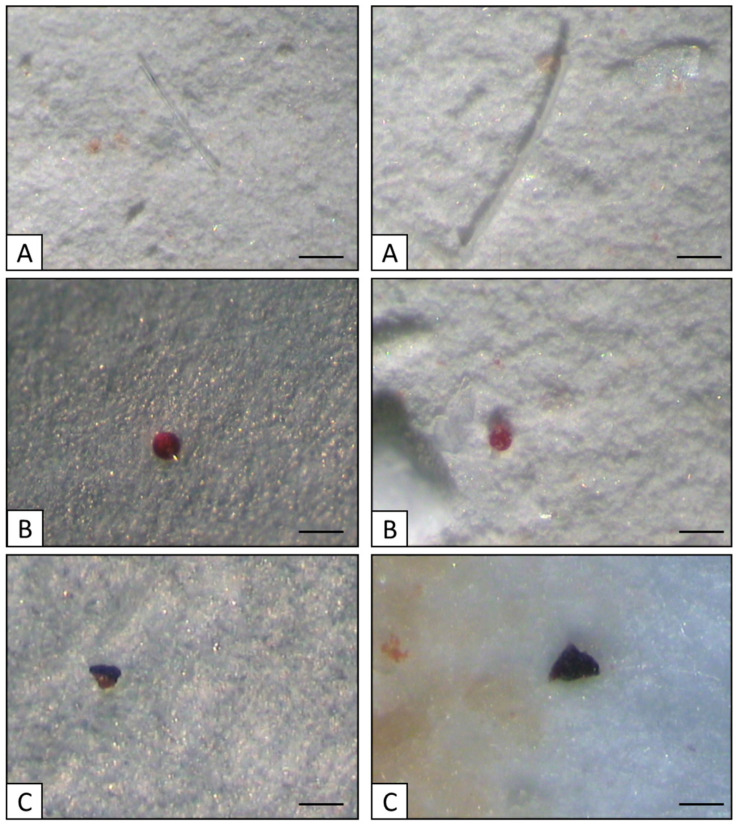
Images of microplastics used in the experiment: fibers (**A**), granules (**B**), and fragments (**C**). (Scale bar: 50 µm).

Finally, filters were observed under a stereomicroscope (Leika MZ6, Leica Microsystem Ltd., Heerbrugg, Switzerland), images were taken using a digital camera (JVC-C1381, JVC, Yokohama, Japan) (Figure 2) and each particle was measured along its longest dimension, using Leica IM500 software (Leica Microsystem Ltd., Heerbrugg, Switzerland). A visual assessment was applied to recognize and classify the spiked microplastics according to the rules of [51] and the hot needle test [52].

The blue cotton fibers produced by the outer clothing worn by laboratory workers were easily recognized during visual microscopy due to their unique color and structure and were subsequently ignored during particle counting.

### 2.4. Gene Expression Analysis

For each sampling time (control group, 0-time group, 2-time group, and 7-time group) five organisms were taken. From each bivalve, separate gills and digestive glands were taken and pools were created, then samples were submitted to biomolecular investigations in order to evaluate a set of target genes, through the analysis of gene expression with RT-qPCR, to evaluate the response of bivalves to stress from microplastic pollutants.

As organs were randomly divided into each pool and sex and gametogenesis status cannot be determined through external morphology of the body, only by histological analysis of the gonads, for this experiment there was no possibility to recognize the sexual traits of each animal belonging to the pool. The molecular analysis was therefore conducted considering the biological responses of the mussel population as a natural collection of both males and females.

The steps performed for the gene expression analysis are as follows: (a) RNA extraction and cDNA synthesis, (b) primers and PCR efficiency (*Eff*); (c) qPCR Sybr Green.

#### 2.4.1. RNA Extraction and cDNA Synthesis

For each sampling time, digestive glands and gills were dissected and quickly snap-frozen in RNA later™ Stabilization Solution (ThermoScientific™ Waltham, MA, USA) and stored individually at −80 °C. Digestive glands and gills were then pooled (4–5 individuals per pool) according to sampling time and, for each pool, total RNA was subsequently extracted using Quick-RNA™MiniPrep Plus kit (Zymo Research, Irvine, CA, USA), following the manufacturer’s recommendations. Briefly, 50 mg of each sample was submerged into 600 µL of DNA/RNA Shield™ and homogenized. For every 300 µL of sample, was added 15 µL Proteinase K and 30 µL PK Digestion Buffer, mixed and incubated at room temperature (20–30 °C) for 5 h. To remove particulate debris, sample was centrifuged and the cleared supernatant was transferred into a nuclease-free tube. An equal volume of RNA Lysis Buffer was added to the supernatant and mixed well. The lysed sample was transferred into a Spin-Away™ Filter and centrifuged at 16,000× g per 30 s to remove the majority of genomic DNA. One volume of ethanol (95–100%) was added to the flow-through and mixed well. Then the mixture was transferred into a Zymo-Spin™ IIICG Column, centrifuged and the flow-through was discarded. An amount of 400 µL RNA Prep Buffer was added to the column, centrifuged and the flow-through was discarded. An amount of 700 µL RNA Wash Buffer was added to the column, centrifuged, and the flow-through was discarded. An amount of 400 µL RNA Wash Buffer was added, centrifuged the column, and the flow-through was discarded. Lastly, 100 µL DNase/RNase-Free Water was added to the column matrix, centrifuged and the eluted RNA was harvested.

RNA extracted from all samples was quantified by the Qubit 2.0 fluorometer (Thermofisher Scientific), using the Qubit™ RNA High Sensitivity (HS) kit, according to manufacturer’s instructions. Concerning purity, all RNA samples showed absorbance ratios (A_260_nm/A_280_nm and A_260_nm/A_230_nm) above 1.9, indicating a high level of purity.

cDNA synthesis reverse transcription (RT) was performed using 2.5 μg of the total RNA using RevertAid H Minus First Strand cDNA Synthesis Kit (Thermo Fisher Scientific) with random hexamers and according to the manufacturer’s instructions. A control cDNA sample was made for each organ by pooling the same volume of each cDNA sample.

#### 2.4.2. Primers and PCR Efficiency (Eff)

The genes to be tested as biomarkers, listed in Table 1, were chosen in agreement with the recent scientific bibliography [34,35,36]. Two genes, β-actin and tubulin, were selected as stable housekeeping already described in the literature by [36].

Primers were synthesized by Eurofins Genomics GmbH (Ebersberg, Germany)). PCR efficiency (*Eff*) was calculated for each primer pair in both organs by making standard curves from serial dilutions of reference cDNA (from 1/50 to 1/800) and using the following formula [53]: Eff = 10^−1/slope^.

Efficiency of the amplification was determined for each primer pair using serial 5-fold dilutions of pooled cDNA and calculated as E = 10 (−1/s), where is the slope generated from the serial dilutions [53]. Primer pairs specificities were checked both in silico and empirically by BLAST analysis and using melting profiles. BLAST analyses indicated all primers were specific, which was confirmed by melting profiles.

#### 2.4.3. qPCR Sybr Green

qPCR Sybr Green was performed using the Applied Biosystems 7500 Real-time System. Assays were performed in triplicate using PowerUp™ SYBR™ Green Master Mix (Thermofisher Scientific) according to the manufacturer’s instructions. Briefly, 2 µL of each sample’s cDNA was used at the concentration of 5 ng. Each PCR reaction (20 µL/well) contained 10 µL of PowerUp™ SYBR™ Green Master Mix (2X), 1.5 µL of forward and reverse primers (500 nM for each primer) and 5 µL of Nuclease-Free Water. The PCR program consisted of 2 min of Dual-Lock DNA polymerase activation at 95 °C followed by 40 cycles of 15 s of denaturation at 95 °C, 15 s of annealing at 55–60 °C and 1 min of elongation at 72 °C. For each sample a melting curve program was performed having the following conditions: 1 cycle 95 °C for 15 s, 60 °C for 1 min, and 95 °C for 15 s. To minimize technical variation, all samples were analyzed on the same run for one gene. Each PCR run included the control cDNA sample and water controls. Relative gene expression was calculated with the 2^−ΔΔCt^ method, extensively used as a relative quantification strategy for quantitative real-time polymerase chain reaction (qPCR) data analysis [54].

### 2.5. Statistical Analysis

The statistical analyses of data were performed using R v4.0.5 (R Core Team, 2021, R Foundation for Statistical Computing, Vienna, Austria) and Excel 2016 (Microsoft, Silicon Valley, CA, USA). Normality of data set was tested with Shapiro–Wilk test. Then, non-parametric tests were used if the data were not normally distributed.

Beta distribution with 95% confidence intervals was used to verify the variability between the three experimental replicates with respect to the percentages of organisms contaminated by microplastics. A Kruskal–Wallis test was also applied to verify any differences between the experimental groups with respect to the variables “size” and “n. particles MPs/g”. When the Kruskal–Wallis test was significant, Dunn’s post hoc tests were carried out for comparisons between all possible pairs, and Bonferroni’s correction was applied.

Linear regression analysis was used to verify the possible existence of a linear relationship between the number of MP particles per gram and time. The Spearman rank correlation test was performed to test any correlation between size of the mussels and the number of MPs.

For the analysis of gene expression data, to assess variations in transcript levels across different sampling times, we employed the non-parametric Mann–Whitney test. The analysis with *p* < 0.05 was considered statistically different.

## 3. Results

### 3.1. MPs’ Accumulation and Depuration

In all experiments conducted in T0 groups, 100% of mussels were contaminated with a number of MPs between 182 and 217 MPs/individuals total number. The average of MPs particles/g ranged from 1.97 to 2.4. In T2 groups, the percentage of contaminated organisms was, respectively, 95, 70, and 75 with a number of MPs between 26 and 63. The average of MPs particles/g ranged from 0.24 to 2.4. In T7 groups, the percentage of contaminated organisms ranged from 55 to 75, the average of MPs particles/g ranged from 0.26 to 1 (Table 2).

By analyzing the percentages of organisms contaminated by microplastics (Table 2), with the relative 95% confidence interval, calculated using the Beta distribution, it is possible to observe for each of the three replicates a variability between the replicates that is not statistically significant, as the 95% confidence intervals are comparable. This means that the presence of the experimental error did not interfere with the results obtained during our experiments.

**Figure 3:**
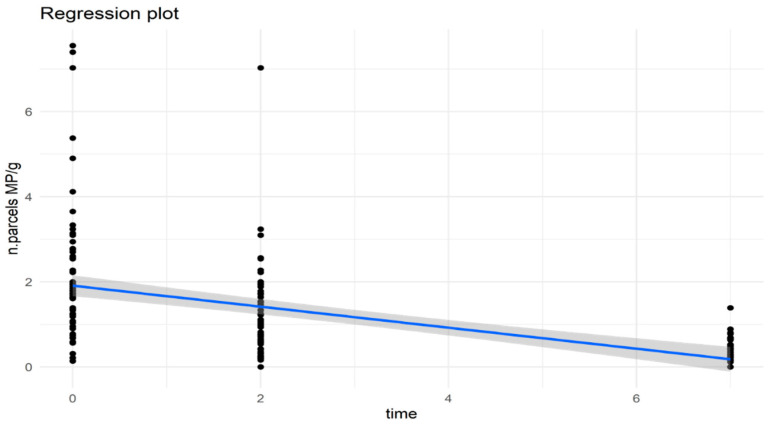
Regression plot of n. particles MPs/g trend in three experimental replicates.

On the values relating to “n. particles MPs/g” a linear regression analysis was performed to verify the possible existence of a linear relationship between the number of MPs particles per gram and sampling times (T0, T2, and T7 group). From the regression plot (Figure 3, Table 3), it is possible to observe a statistically significant decrease (*p* Value 2.5 × 10^−14^) in the number of MPs found per gram of soft tissue of the analyzed mussels after 7 days of depuration process (group T0: average 2.17 MPs/g; group T2: average 0.49 MPs/g; group T7: average 0.27 MPs/g). This decrease, although already present after 2 days, was not statistically significant, but instead, it became so after 7 days of depuration.

**Figure 4:**
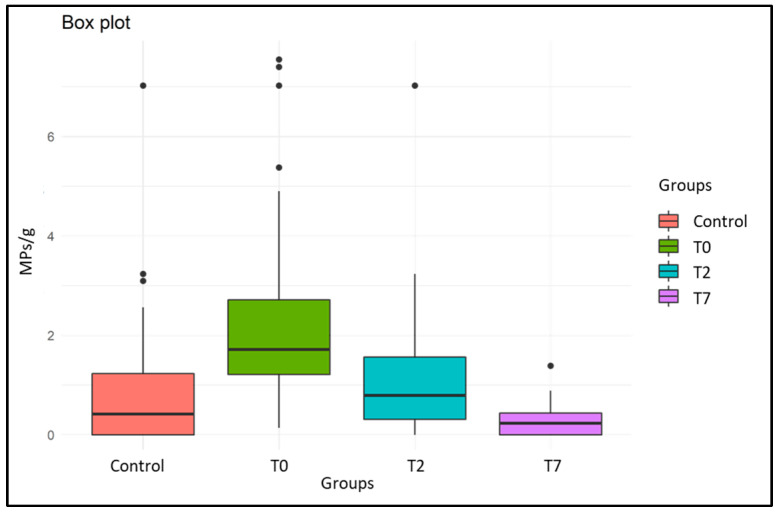
Box plot representing the number particles MPs/g differences in experimental groups. Control group: organisms collected at the end of the 7 days of acclimatization phase; Group T0: organisms collected at the end of the 3 days of exposure to MPs; Group T2: organisms collected at the end of the 2 days of depuration (microbiological depuration); Group T7: organisms collected at the end of the 7 days of depuration (experimental depuration for MPs).

Furthermore, to verify the differences in the experimental groups (control, T0, T2, and T7 group) with respect to the variable “*n. particles MPs/g*” (Figure 4), the Kruskal–Wallis test was performed, followed by related Dunn’s post hoc tests (with Bonferroni correction). This comparison showed a statistically significant difference between: (a) the control group vs. T0 group (*p* Value: 1.23336 × 10^−6^), indicating that T0 group was correctly contaminated experimentally with MPs compared to the control group; (b) T0 group vs. T7 group (*p* Value: 0.007125783), highlighting a good capacity of the mussels after 7 days of depuration to properly remove the microplastics accumulated within their soft tissue.

For the variable “size” of the MPs (Table 4), the statistical analysis carried out on the experimental groups highlighted statistically significant differences between the control group vs. T0 group (*p* Value: 4.42 × 10^−7^) and control group vs. T2 group (*p* Value: 0.0001). This comparison suggests that the frequency in the control group of microplastic particles of size 50–100 µm (most represented size class) is statistically different from that found in groups T0 (most present size class: 1000–2000 µm) and T2 (class most present dimensional: <1000 µm). In addition, statistically significant differences were found between the T0 group vs. T7 group (*p* Value: 0.007048), pointing that the frequency in the T0 group of MPs of size 1000–2000 µm (most represented size class) is statistically different from that present in the T7 group (most present size class: <1000 µm).

**Figure 5:**
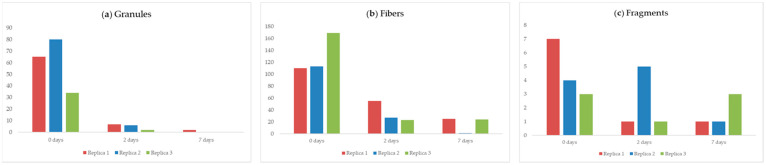
Graphical representation of the number of granules (**a**), fibers (**b**), and fragments (**c**) in the three experimental replicates. *Error bars = Standard deviation*.

The results of the variable “type of microplastics” is reported in Figure 5, which shows the different decrease for each type of microplastic chosen in the three experimental replicas. Granule seems to be eliminated more effectively by mussels, both after 2 and 7 days of depuration, followed by filament and fragment. 

Finally, the Spearman’s rank correlation rho test was performed to evaluate a possible positive correlation between the size of mussels (expressed in soft tissue weight) and the number of microplastic particles found. The results indicate that there was no significant correlation between the two variables (*p* Value = 0.6313).

### 3.2. Gene Expression Analysis

Molecular biomarkers, already known from other studies to be involved in the response to pollutants [34,35,36] were tested in analyzed mussels’ gills and digestive glands. The digestive gland and gills are both tissues of relevant interest for the analysis of the change in the expression of target genes. The first has been described as the organ in which pollutants accumulate in higher concentrations, while gills are the dominant site of interaction with the environment [36].

**Figure 6:**
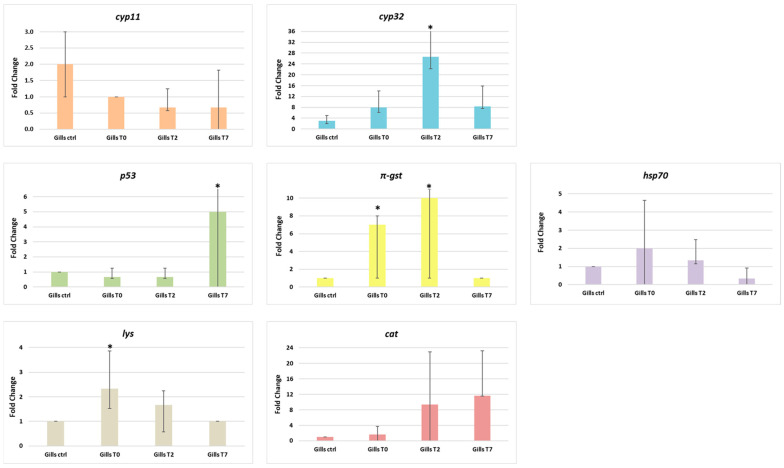
Gene expression results calculated with the 2^−ΔΔCT^ method [54]. Bar graph presentation of the fold change in target genes analyzed (*cyp11*, *cyp32*, *p53*, *π-gst*, *hsp70*, *lys*, and *cat*) as a function of sampling times in gills of *M. galloprovincialis* after 3 days MP exposure phase (Gills T0), after 2 days depuration phase (Gills T2) and after 7 days depuration phase (Gills T7). All RT-PCR results are normalized to β-actin and tubulin, the housekeeping genes, and expressed as change from their respective controls. The average values were obtained from three experiments. Significant difference < 0.05 is indicated by an asterisk. Values represent the means ± standard deviation (SD).

Overall, a differential expression was observed (Figure 6) between the groups of mussels tested, in particular, most of the differences were found in the gills, thus defining it as the target organ for the use of these biomarkers [34,55].

In gills, a significant increase (*p* = 0.001) in mRNA abundance of *cyp32* was found after 2 days of depuration (Gills T2) when compared to the control group, whereas *cyp11* mRNA levels were basically unaltered in relation to the control group, as described by [34].

Transcriptional levels of the p53 gene in gills significantly increased (*p* = 0.001) in mussels after 7 days of depuration (Gills T7) compared to the control group, whereas mRNA levels of π-gst significantly increased (*p* = 0.001) in mussels both after 2 and 7 days of depuration (Gills T2 and Gills T7). In the gills, the expression levels of *hsp70* were upregulated in the mussels exposed (Gills T0) in comparison to the mussels subjected to 7 days of depuration (Gills T7).

**Figure 7:**
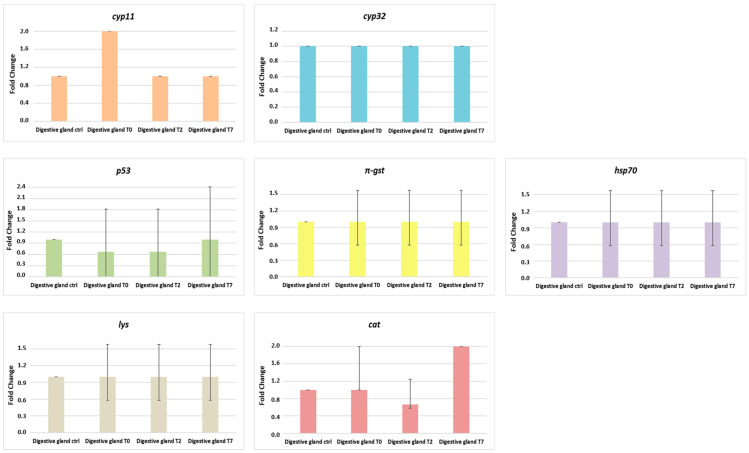
Gene expression results calculated with the 2^−ΔΔCT^ method [54]. Bar graph presentation of the fold change in target genes analyzed (*cyp11*, *cyp32*, *p53*, *π-gst*, *hsp70*, *lys*, and *cat*) as a function of sampling times in digestive gland of *M. galloprovincialis* after 3 days MP exposure phase (Digestive gland T0), after 2 days depuration phase (Digestive gland T2) and after 7 days depuration phase (Digestive gland T7). All RT-PCR results are normalized to β-actin and tubulin, the housekeeping genes, and expressed as change from their respective controls. The average values were obtained from three experiments. Significant difference <0.05 is indicated by an asterisk. Values represent the means ± standard deviation (SD).

Finally, always in gills, mRNA abundance of the lys gene in mussels exposed (Gills T0) is statistically upregulated (*p* = 0.002), whereas the expression levels of the cat gene showed no significant increase in both groups subjected to depuration compared to the control group. Concerning the target genes’ expression in the digestive glands of *M. galloprovincialis* (Figure 7), our results highlight no statistically significant increase in the level of mRNA of *cyp11* in the digestive glands of mussels exposed for 3 days to MPs (Digestive gland T0) compared to mussels subjected to depuration. Furthermore, in the digestive gland, transcriptional levels of genes *cyp32* e *π-gst*, associated with biotransformation and detoxification processes, were unaltered in all mussel groups.

Regarding the p53 gene, the expression was slightly decreased in the digestive gland of mussels after both the exposure phase (Digestive gland T0) and 2 days of depuration (Digestive gland T2) compared to the control group. Cell tissue repair-related gene *hsp70*, presented an unaltered transcriptional level in the digestive gland of all mussels groups.

Lastly, regarding immune genes in the digestive gland, mRNA levels of *lys* were unaltered, whereas the transcriptional levels of the *cat* gene appeared upregulated in the depurated group (Digestive gland T7) when compared to other groups.

## 4. Discussion

Exposure experiments are effective methods to study the uptake of contaminants. The ingestion and biological effects of microplastics have been tested in numerous previous studies [12,33,38,39,40,42,43,52,56,57]. Considering the limitations imposed by the common approach of using a single type of microplastic, the present study instead used three types of microplastic particles (i.e., fibers, fragments, and granules) usually found in the marine environment to simulate as much as possible the microplastic pollution [45,46]. This approach allows us to obtain more detailed information about MP accumulation and depuration processes in mussels.

In our exposure experiments, we tried to recreate a condition of microplastic pollution, using the main types of microplastics found in seawater columns with a final concentration frequently reported in the marine environment, however, we encountered limitations given by our experimental design. This could be due to (a) microplastics present in marine environments could have different physical–chemical properties such as shapes, sizes, colors, compositions, and additives [58]; (b) microplastic accumulation in the marine environment occurs over a long period while the exposure experiments are shorter [20]. Therefore, great efforts are needed to simulate these aspects in exposure studies [20].

The linear regression analysis used to verify a possible relationship between the variables “number of MPs particles per gram” and “time” showed considerable variability in terms of the presence of microplastics in mussels’ soft tissue at the end of the 3-day exposure phase (T0). This variability confirmed by a low corrected R-squared value (0.275) could be explained by too short contamination times, so that all mussels did not bio-accumulate microplastics uniformly. In fact, our observations are in agreement with other studies [42] where short exposure times (10, 20, and 40 min) were performed and a higher number of microplastics, used for contamination, was found in the exposure water when compared to those found in bivalves’ soft tissue.

The mussels were able to remove most MPs within 7 days, with about an 80% reduction in average MPs’ concentration occurring between days 0 and 7, highlighting a statistically significant decrease (*p* Value 2.5 × 10^−14^) in microplastic particles found per gram in analyzed mussels’ soft tissue. Our results are in agreement with previous studies [5,33] performed on both mussels and oysters, which demonstrated that a longer purification time (5–7 days) could give a significant reduction in MPs’ concentration of bivalves intended for human consumption. An example is the study conducted by Coverton et al. [5], which observed in specimens of *Crassostrea gigas*, 5 days after the start of the depuration process, a reduction in the concentration of MPs’ particles equal to 73%. Comparing T0 and T2 groups (mussels at the end of 2-day depuration), where we found 2.17 MPs/g and 0.49 MPs/g, respectively, no statistically significant differences emerged (*p* Value 1). This result suggests that the two-day depuration time is not long enough to ensure the complete elimination of microplastic particles. Similarly, a previous study conducted by [59] highlights that differences in depuration rates among various particles probably reflect the differences in particle fate within bivalves. There is a fast elimination (between 0 and 1 h of depuration process) of those particles associated with the gills, or those recently rejected by the gills and labial palps and incorporated into mucous for ejection as pseudofeces. There is, instead, a long retention time for particles associated with a variety of tissues, including the digestive tract, mantle, and muscle [59].

Other studies [39] also suggest that the short depuration times are not sufficient to completely eliminate MPs in bivalves. In addition, longer depuration times (7 days) have been tested in other studies [60,61] suggesting that even a longer depuration period would not be enough to completely remove MPs accumulated in bivalves. Therefore, it is noteworthy that depuration can minimize the effects caused by MP contamination, even if it does not reach a 100% reduction [39].

Concerning microplastics’ size class found in analyzed mussels, our results showed a statistically significant difference between the T0 and T7 groups (*p* Value: 0.007048). In the T0 group, the most represented size class was 1000–2000 µm, unlike in the T7 group where microplastics <1000 µm in size were more present. These results could be explained by the ability of bivalves to eliminate larger microplastic particles more easily and in a shorter time. In fact, even in other bivalves, such as *Mytilus edulis* and *Crassostrea gigas*, a greater depuration capacity of larger microplastic particles has been found [38,39,40,41,42,43,44,45,46,47,48,49,50,51,52,53,54,55,56,57,58,59,60,61,62]. On the other hand, Fernandez and Albetosa [33] demonstrated that mussels (*M. galloprovincialis*) were unable to eliminate smaller particles (<6 μm) as well as larger ones, which remained in digestive glands.

The type of microplastics that seems to be eliminated more effectively by mussels, both after 2 and 7 days of depuration, are granules, followed by filaments and fragments. Probably granules’ spherical shape allows them to be less retained within the mussels, and are therefore able to get rid of them more easily [39,40,41,42,43,44,45,46,47,48,49,50,51,52]. In fact, we observed an almost total absence of this type of microplastic in all analyzed organisms after 7 days of depuration.

Fibers instead, due to their geometry, may be better trapped in mussels’ gills and digestive glands, resulting in a longer depuration time compared with other microplastic types [23,39,52,63,64]. Regarding the fragments, we did not observe a typical trend during the overall duration of the experiment. Furthermore, no significant correlation was highlighted between variables “mussels’ weight” and “number of MPs particles per gram”. As also reported in other experiments, mussels’ ability to accumulate microplastics, as well as to eliminate them, is not strictly correlated with the organisms’ size [39]. Other studies, instead, showed a negative correlation between microfiber levels and mussel weight, which could be explained by the fact that in the Mytilus species, pumping and filtration rates decrease with higher soft tissue mass [65,66].

In addition to MPs’ bioaccumulation and depuration experiments, seven biomarkers, known to be involved in pollutant response [34,35,36] were tested in *M. galloprovincialis’* gills and digestive glands.

A differential gene expression was observed between the groups of mussels tested and in particular, most of the differences were found in the gills, thus defining it as the target organ for the use of these biomarkers. Indeed, the gills are a key organ for the uptake during filtration processes of pollutants present in the marine environment and are known to temporarily accumulate the contaminants before their probable transfer to the digestive gland and other tissues [67].

Our results give further proof of the involvement of the *cyp32* gene in mussel biotransformation processes [34,35,36,37,38,39,40,41,42,43,44,45,46,47,48,49,50,51,52,53,54,55,56,57,58,59,60,61,62,63,64,65,66,67,68] and of its potential as a biomarker since we observed a significantly higher *cyp32* mRNA level in the gills of mussels after 2-day depuration (T2) compared to the control group.

Variations of expression were observed for the *p53* gene, which plays an important role in apoptosis signaling [55] and therefore it is considered a cellular stress marker in mussels [69]. In particular, *p53* transcriptional levels in contaminated mussels (T0) showed a downregulation compared to other groups. This data could highlight, as described by Lacroix et al. (2014) [34], that inhibition of apoptosis processes is activated in contaminated mussels rather than an enhancement, most probably due to the existence of a powerful anti-apoptotic system in bivalves. This could explain their high resilience to pollution as suggested by Wang et al. [70].

Concerning the *π-gst* isoform, which plays a role in the antioxidant defense system [34], a statistically significantly higher mRNA level was observed in the gills exposed to MPs for 3-day mussels (T0) compared to other mussels. Also, other authors [34,71] observed in general higher π-gst mRNA levels in the gills of mussels sampled in polluted sites compared to control sites, which highlights the potential role of *π-gst* as a pollution biomarker. This same gene, instead, in the digestive glands of *M. galloprovincialis,* shows unaltered transcriptional levels, which has also been described by Brandts et al. [36] and Zanette et al. [68].

In gills, the observed increased expression of the *hsp70* gene after the exposure phase (T0) has also been described in other studies regarding the contamination with MPs [36,71,72], and it may indicate an induction of de novo synthesis of these proteins as an attempt to cope with pollutants.

Concerning the gene *cat*, our results showed an upregulation of this gene in the two mussel groups subjected to the two depuration times (T2 and T7). As reported by other recent studies [36,73,74], an upregulation of the *cat* gene transcript after exposure to MPs suggested that this cathepsin would play a role in an intracellular apoptotic pathway.

Lastly, the mRNA levels of *lys* noticeably increased in in mussels’ group exposed to MPs for 3 days (T0), as also described by Brandts et al. [36], whose study reported such an increase in nanoplastic-exposed organisms.

## 5. Conclusions

Our evidence suggests that depuration processes can significantly reduce MP contamination in *M. galloprovincialis*, even if, as also indicated by Covernton et al. [5], it would be difficult to completely remove all MPs from mussels under commercial depuration conditions. Indeed, in order to obtain a significantly reduced MP contamination in bivalves it is necessary (a) to use a “clean” facility (filtering water, covering tanks, and longer depuration time); and (b) to practice a depuration process for longer times than those already in use (Reg. UE 627/2019). Unfortunately, a longer depuration period (5–7 days) could implicate more cost to the industry, and consequently increase shellfish’s cost to the consumer [5]. Therefore, it is necessary to conduct more research to refine the depuration time that allows for good results in terms of cost/benefit.

Concerning molecular biomarkers analysis, several genes were found differentially expressed between sample mussel groups. However, further studies are necessary to confirm the diagnostic ability of this set of biomolecular biomarkers in marine environmental monitoring programs to be used as a supplement to traditional chemical and biomarker measures. Furthermore, it is known that sex and gametogenesis cycle could influence contaminant uptake and elimination or biomarkers levels in molluscs [75,76,77], as well as the Mytilus gene expressions involved in natural biological rhythms [78] or when the animals are exposed to endocrine disruptors [79,80,81]. Considering this, further investigations are needed involving histological observation of gonadal samples alongside gills and digestive glands, in order to analyze whether these molecular responses would be influenced by sex or the gametogenesis cycle in which the animals are found.

## Figures and Tables

**Table 1:** Gene name, abbreviation, pathway/function, primer pair, GenBank accession numbers, and references, for target genes analyzed in digestive gland and gills of *Mytilus galloprovincialis*.

Gene Name	Abbreviation	Pathway/Function	Forward (5′-3′)	Reverse (5′-3′)	Accession Number	Reference
β-Actin	*act*	Housekeeping gene	CGACTCTGGAGATGGTGTCA	GCGGTGGTTGTGAATGAGTA	AF157491.1	[36]
α-Tubulin	*tub*	Housekeeping gene	CTTCGGTGGTGGTACTGGAT	AGTGCTCAAGGGTGGTATGG	HM537081.1	[36]
Cytochrome P450-1-like-1	*cyp11*	Phase I biotransformation	TGGTTGCGATTTGTTATGCCCTGGA	GGCGGAAAGCAATCCATCCGTGA	JX885878	[34]
Cytochrome P450-3-like-2	*cyp32*	Phase I biotransformation	CAGACGCGCCAAAAGTGATA	GTCCCAAGCCAAAAGGAAGG	AB479539	[34]
π-glutathione-S-transferase	*π-gst*	Phase II biotransformation	CCTGAAACCAACCAAGGGTTACAT	TGGACTCCTGGTCTAGCCAACACT	AF227977/AF527010	[34]
*p*-53 tumor suppressor-like	*p53*	Cellular stress response	CAACAACTTGCCCAATCCGA	GGCGGCTGGTATATGGATCT	AY579472/ DQ158079	[34]
Heat shock protein 70	*hsp70*	Cellular tissue repair	CCCTTTCTTCAAGCACACAAGCA	AACTGGTTCCATGGTTCCTCTGAA	AF172607	[36]
Cathepsin	*cat*	Immune system	CGCAGCTAATGTTGGCGCC	CTACGGCGATTGGTCCCTG	AF172607	[36]
Lysozyme	*lys*	Immune system	TCGACTGTGGACAACCAAAA	GTGACCAATGTACCTCGCCA	AF334662/AF334665	[35]

**Table 2:** Summary table of the bivalves contaminated by MPs in the three experimental replicates with u.c.l. (upper control limit) and l.c.l. (lower control limit) at 95%. Group T0: 20 organisms collected at the end of the 3 days of exposure to MPs; Group T2: 20 organisms collected at the end of the 2 days of depuration (microbiological depuration); Group T7: 20 organisms collected at the end of the 7 days of depuration (experimental depuration for MPs).

	N. Organisms Found Contaminated	N. Organisms Found Contaminated (%)	l.c.l.	u.c.l.	N. MPs/Individuals’ Total Number ± SD	Average MPs Particles/g ± SD	Granules (%)	Fibers (%)	Fragments (%)
EXPERIMENTAL GROUPS (replicate 1)									
Group T0	20/20	100%	87%	100%	182 ± 8.69	2.40 ± 1.10	36	60	4
Group T2	19/20	95%	76%	99%	63 ± 2.40	0.79 ± 0.64	11	87	2
Group T7	15/20	75%	53%	89%	28 ± 1.23	0.39 ± 0.35	7	89	4
EXPERIMENTAL GROUPS (replicate 2)									
Group T0	20/20	100%	87%	100%	217 ± 5.58	2.15 ± 1.24	37	61	2
Group T2	14/20	70%	48%	85%	38 ± 1.76	0.45 ± 0.31	16	71	13
Group T7	13/20	65%	43%	82%	20 ± 0.97	0.17 ± 0.15	0	95	5
EXPERIMENTAL GROUPS (replicate 3)									
Group T0	20/20	100%	87%	100%	203 ± 8.29	1.97 ± 1.32	17	83	0
Group T2	14/20	95%	48%	85%	26 ± 8.19	0.24 ± 0.11	8	88	4
Group T7	11/20	55%	34%	74%	27 ± 0.78	0.26 ± 0.20	0	89	11

**Table 3:** Table of coefficients of the linear regression analysis to verify a linear relationship between the “n. particles MPs/g” and the “time expressed in days”.

	Coefficients	Standard Error	Stat t	Significance Value	Lower 95%	Upper 95%
Intercept	1.91	0.12	15.28	3 × 10^−34^	1.66	2.16
TIME	−0.25	0.03	−8.30	2 × 10^−14^	−0.31	−0.19

**Table 4:** Summary table of the percentage presence of the various size classes of microplastic particles in the comparison groups (control, T0, T2, and T7 groups).

	10–50 µm	50–100 µm	200 µm	100–500 µm	<1000 µm	1000–2000 µm	2000–3000 µm	>3000 µm
Control group	1%	70%	0%	1%	15%	7%	3%	3%
T0 group	0%	0%	6%	1%	18%	43%	24%	7%
T2 group	2%	13%	2%	0%	33%	17%	31%	2%
T7 group	1%	5%	0%	3%	51%	20%	11%	9%

## Data Availability

Data are presented in this article in the form of figures and tables.
